# Personality, sperm traits and a test for their combined dependence on male condition in guppies

**DOI:** 10.1098/rsos.220269

**Published:** 2022-06-01

**Authors:** Edward Galluccio, Rowan A. Lymbery, Alastair Wilson, Jonathan P. Evans

**Affiliations:** ^1^ Centre for Evolutionary Biology, School of Biological Sciences, University of Western Australia, 6009 Western Australia, Australia; ^2^ Centre for Ecology and Evolution, University of Exeter, Cornwall Campus, Penryn, UK

**Keywords:** behavioural syndrome, animal personalities, multivariate selection

## Abstract

There is evidence that animal personality can affect sexual selection, with studies reporting that male behavioural types are associated with success during pre- and post-copulatory sexual selection. Given these links between personality and sexual traits, and the accumulating evidence that their expression can depend on an individual's dietary status (i.e. condition), a novel prediction is that changes in a male's diet should alter both the average expression of personality and sexual traits, and their covariance. We tested these predictions using the guppy *Poecilia reticulata*, a species previously shown to exhibit strong condition dependence in ejaculate traits and a positive correlation between sperm production and individual variation in boldness. Contrary to expectation, we found that dietary restriction—when administered in mature adult males—did not affect the expression of either behavioural (boldness and activity) or ejaculate traits, although we did find that males subjected to dietary stress exhibited a positive association between sperm velocity and boldness that was not apparent in the unrestricted diet group. This latter finding points to possible context-dependent patterns of covariance between sexually selected traits and personalities, which may have implications for patterns of selection and evolutionary processes under fluctuating environmental conditions.

## 1. Introduction

There is growing interest in understanding the reproductive fitness consequences of animal personalities [1], defined as individual differences in behavioural traits that are consistently expressed across contexts and/or time [2,3]. In particular, attention has focused on possible associations between behavioural traits such as boldness, exploration tendency and aggressiveness and those that determine access to mating opportunities, such as the ability to attract or compete effectively for mates [4]. Bolder or highly aggressive males, for instance, may be better able to defend an area from rivals [5–7], while individual plasticity in exploration tendency can explain variance in a male's lifetime reproductive success [8]. Although there has been less attention on linking animal personality research to post-copulatory sexual selection (i.e. sperm competition, where ejaculates from different males compete for fertilizations [9], and cryptic female choice, where females influence the outcome of such contests [10]), recent work has reported that male behavioural traits such as boldness and exploration tendency can predict individual variation in sperm traits [11] and sperm competition intensity [12]. Together, this accumulating body of evidence suggests that male personality may play a role during both pre- and post-copulatory episodes of sexual selection, with potentially far-reaching fitness implications in polygamous species.

Many sexually selected traits depend on an individual's condition, which can be conceptualized broadly as the pool of resources allocated to traits that enhance fitness [13,14]. Evidence for the condition-dependent expression of pre- and post-copulatory sexual traits is widespread (e.g. reviewed in [13,15]), and there is putative evidence that behavioural traits can similarly exhibit some degree of condition dependence. For example, an individual's willingness to explore novel and potentially hazardous environments (i.e. boldness) can reflect its internal ‘state’ (e.g. [16]), while dietary history can influence both among-individual variation (personality) and within-individual variation (behavioural plasticity) in a range of personalities, including aggression, mating activity and exploration behaviour [17,18]. Given this possible shared dependence of both personality and sexually selected traits on condition, and the aforementioned relationships between (male) personality and secondary sexual traits, one expectation is that changes to an individual's condition should simultaneously impact both personality and reproductive traits and their potential covariances. Indeed, there is growing evidence that correlations among traits can change along environmental gradients (e.g. degree of stress; see reviews in [19,20]), and therefore we might expect that changes in the level of resources available for allocation to fitness-enhancing traits will alter the strength of relationships between personality and sexual traits. Despite this prediction, we know of no studies that have experimentally tested for condition-dependent relationships between personality and those involved in reproduction.

Here we test whether the experimental manipulation of male condition influences the expression of personalities, sperm traits and their possible covariances in the guppy *Poecilia reticulata*. Guppies are ideal models for such an investigation, given the prior extensive knowledge of sexual selection in this species [21,22], a large body of animal personality research that has confirmed that individuals vary along behavioural axes of ‘boldness–shyness’ and ‘activity–inactivity’ (e.g. [23]), and recent evidence for an association between male personalities and sperm traits [11]. Specifically, Gasparini *et al.* [11] reported a positive correlation between a male's tendency to emerge from a refuge (i.e. boldness) and sperm production. Moreover, there is extensive prior evidence that dietary restriction causes a decline in both the number and composition of sperm produced by male guppies [24–26] and therefore we have an *a priori* expectation that changes in male condition induced through changes in diet should simultaneously impact both ejaculate traits and the behavioural (personality) traits with which they are correlated. We, therefore, predicted that by inducing dietary stress we would cause a simultaneous reduction in the expression of male behavioural traits such as boldness and ejaculate traits. Furthermore, we test whether any relationship between personalities and sperm is contingent on male condition, given the expectation that patterns of covariation between physiological traits and behaviour can be sensitive to the level of environmental stress [19,20].

## 2. Material and methods

### 2.1. Study species and its maintenance

Experimental fish were obtained from a mixed-sex stock population maintained at UWA under standard laboratory conditions (approx. 1 : 1 sex ratio; 26 ± 1°C; fed ad libitum a combination of live *Artemia nauplii* and commercial dry food). These stocks were descendants of fish caught from a feral population in the Alligator Creek river in Queensland, most likely originating from introduced fish from a natural population in Guyana [27]. Like other poecilid fishes, guppies are livebearers with internal fertilization, and males transfer spermatozeugmata (unencapsulated sperm bundles) to females at mating. Males and females become sexually differentiated from five weeks of age and are sexually mature by approximately 3–4 months of age [22,28]. The males used in this experiment were aged approximately six months and were therefore fully sexually mature at the onset of the feeding and behavioural trials. Male guppies exhibit a spermatogenic cycle of approximately 36 days [29], and therefore our dietary treatments, which lasted one month (see below), would have impacted almost all critical phases of developing sperm.

### 2.2. Experimental design

Experimental males (*n* = 56) were removed from mix-sex stock aquaria and assigned haphazardly into one of two diet treatments, hereafter termed ‘high quantity’, which effectively corresponds with the feeding conditions (ad libitum) experienced during their early rearing, and ‘low quantity’, for one month, which has been shown to be long enough to induce significant phenotypic effects on sperm in guppies [30]. Throughout the feeding trials, fish were housed individually in 2 l plastic containers in a controlled-temperature room (26°C). Both treatment groups were fed dry fish feed (300–500 µm pellets provided by NutraKol Pty Ltd, Western Australia) once daily, 5 days a week. Following the methodology used for diet manipulation in previous studies on guppies (e.g. [25]), the ‘high quantity’ treatment group was fed at a rate of approximately 4% of their body weight (1.9 mg) while the ‘low quantity’ treatment group was fed at a rate of approximately 2% of their body weight (0.9 mg). The positioning of the tanks on the shelves with respect to treatment status was alternated, and opaque screens were placed between adjacent tanks to avoid behavioural interactions between test subjects. To ensure that the males were stimulated to produce sperm throughout the course of the study, female guppies were placed within view of each tank, as male guppies remain sexually active in the presence of females [31,32]. Due to logistical constraints, eight fish were tested per week, with a total of seven such weekly ‘blocks’. The fish within each block were divided evenly across the two treatment groups. The tanks were illuminated by TLD 36 W fluorescent lamps on a 12 : 12 h lighting schedule.

### 2.3. Behavioural trials

Personality assays involved an emergence test and an open-field test (see [11]). In order to mitigate possible effects of hunger on a male's performance during the behavioural assays, all males were fed at a rate of 4% of their body weight on the day of their behavioural assay. For the emergence test, each fish was placed initially inside an uncovered circular refuge of opaque PVC (10 cm diameter), which itself was placed within a circular arena (40 cm diameter). A video camera (StreamCam, Logitech International SA, Switzerland) was secured above the arena to allow the fish's movements to be observed without human disturbance. After a 5-minute period, during which the fish was allowed to acclimatize to its new surroundings, the door of the refuge was gently pulled away, allowing the fish to emerge. Using video footage, the time (in seconds) taken by an individual to emerge from the refuge was recorded. If the individual emerged within 10 min, its behaviour while moving around the arena was then observed over an additional 5-minute interval. The time (in seconds) the individual spent inactive or ‘frozen’, as opposed to actively moving around, was measured over this period. As with Gasparini *et al.* [11], the data for emergence time and time spent frozen were then inverted to provide final scores for ‘boldness’ and ‘activity’, respectively. For example, any individuals that failed to emerge from the refuge over the 10-minute interval received a boldness score of 0 and an activity score of NA (not observed). To test whether the behaviour was repeatable, each individual was subjected to two of these behavioural assays. The assays for five of the blocks were conducted two days apart, with the one-day gap serving to ensure the test subjects were given sufficient time to recover from the experience [33]. However, due to logistical constraints, in two of the blocks the trials were conducted on consecutive days.

### 2.4. Sperm analysis

Sperm traits were assayed immediately after males completed their final behavioural assay. The males were first anaesthetized in AQUI-S before being placed under a dissecting microscope on a glass slide. Their intromittent organ (gonopodium) was then swung forward beyond 90° and 20–60 µl of an extender medium (207 mM NaCl, 5.4 mM KCl, 1.3 mM CaCl_2_, 0.49 mM MgCl_2_, 0.41 mM MgSO_4_, 10 mM Tris, pH 7.5) was placed at its base using a pipette. The extender medium was used to prevent the sperm bundles from breaking apart before they could be analysed [34]. Light pressure was then applied to the male's abdomen to expel sperm [35]. Two aliquots, each of 2 µl and containing 2–3 spermatozeugmata, were then collected from the sperm pool and each placed in a separate chamber of a 12-well multitest slide for analysis (MP Biomedicals, Aurora, OH, USA). We then added 3 µl of 150 mmol l^−1^ KCl to each well to activate the sperm [36]. The remainder of the ejaculate was collected from the slide and placed in an Eppendorf tube with a known volume of extender medium for subsequent sperm counts.

Sperm velocity was estimated through computer-assisted sperm analyses (CASA; CEROS sperm tracker, Hamilton-Thorne Research, Beverly, MA, USA), which generated several measures of sperm swimming speed, including the average path velocity (VAP), straight-line velocity (VCL) and curvilinear velocity (VCL). Here, we focus on VCL, which describes the actual velocity of sperm cells across the track, as this parameter is known to predict competitive fertilization success in guppies [37]. Sperm velocity measures were based on an average of 419.69 **±** 25.25 s.e. sperm tracks per sample (mean value is taken for *n* = 45 males; *n* = 8 males did not produce sufficiently motile sperm for CASA). Sperm counts were estimated from the reserved ejaculate samples, which were vortexed for 30 s before being counted using an Improved Neubauer hemocytometer [38].

### 2.5. Data analysis

We analysed all data in R v. 3.6.3 [39]. Prior to analyses, personality and sperm traits were scaled to standard deviation units, to allow comparisons of effect sizes from univariate models, and fitting of multivariate models [40].

#### 2.5.1. Univariate analyses

First, we fitted linear mixed-effects models using ‘lme4’ [41] to explore univariate effects of diet treatment on each trait. All models included a fixed effect of diet treatment and a random effect of block. There was significant among-block variance in boldness (likelihood ratio test, LRT; *G*^2^ = 5.74, *p* = 0.017) but not any other trait (LRTs; all *p* > 0.18). Inclusion or exclusion of block did not affect the results of the diet treatment for any trait (electronic supplementary material). In addition to the above effects, the models for behavioural traits (boldness and activity) included random effects of male ID (to partition within- from among-individual variance associated with the repeated measures structure) and a fixed effect of assay number (i.e. whether the observation was from the first or second behavioural assay for each individual). The individual repeatability of behavioural traits, comparing among-male variance to within-male variance, was calculated using Gaussian mixed models and LRTs in ‘rptR’ [42]. Because variances have a lower bound at zero, *p-*values are calculated for LRTs in ‘rptR’ by assuming the difference in log-likelihoods follows a mixture *χ*^2^ distribution with one degree of freedom and point mass at zero [43]. Significance of the fixed diet treatment effect for each trait was tested with Wald *χ*^2^ tests.

#### 2.5.2. Multivariate analyses

Second, we extended our analysis to a multivariate framework to test for (a) associations among the personality (boldness and activity) and ejaculate (sperm number and sperm velocity) traits and (b) an effect of diet treatment on the strength of any associations among traits. To these ends, we fitted a multivariate linear mixed-effects model on the four response traits using ASreml-R v. 4.1.0.154 [44], following Houslay & Wilson [45]. We set the random effect structure to allow partitioning of within- and among-individual variances and correlations to be estimated for the behavioural traits, which had repeated measures for each male. We constrained the within-individual variances and correlations involving sperm traits, which had a single measure per male, to 1 × 10^−8^ and zero, respectively (as correlations can be zero but variances must be a positive number [45]). We included a fixed effect of treatment on the full multivariate response and a fixed effect of assay number for boldness and activity. We tested the significance of fixed effects on overall multivariate phenotype with conditional Wald F tests, and the significance of among-individual correlations between traits by re-fitting the model with among-individual correlations fixed to zero and conducting LRTs against the full model. Lastly, to determine whether diet treatment affected among-individual correlations between traits, we extracted the correlation coefficients (*r*) and associated standard errors (SE) within each diet treatment (high and low). We applied a parametric bootstrap following Morrissey *et al*. [46] to estimate distributions of the difference between pairwise correlation coefficients in each treatment. Significances of correlations within each diet were calculated using LRTs as described above for the full dataset.

## 3. Results

### 3.1. Univariate analyses

There were no significant effects of diet on any individual traits in univariate mixed-effects models (table 1). Boldness was marginally higher in the first assay repeat than the second ($\chi_{1}{\;}^{2}=3.71$, *p* = 0.054), while activity was significantly higher in the first assay than the second ($\chi_{1}{\;}^{2}=18.67$, *p* < 0.001). Overall, repeatability of boldness for individual males was marginally non-significant (*R* = 0.18 ± 0.12 s.e.; LRT of among-male variance, $\chi_{1}{\;}^{2}=2.41$, *p* = 0.060), while activity was significantly repeatable for individual males (*R* = 0.54 ± 0.14 s.e.; LRT, $\chi_{1}{\;}^{2}=10.10$, *p* = 0.001). When tested within each diet treatment, there was significant repeatability in boldness for high diet males (*R* = 0.46 ± 0.19 s.e.; LRT, $\chi_{1}{\;}^{2}=4.39$, *p* = 0.018) but not for low diet males (*R* = 0.10 ± 0.13 s.e.; LRT, $\chi_{1}{\;}^{2}=0.41$, *p* = 0.260). While this is suggestive of higher behavioural consistency (measured by *R*) in the high diet treatment, we do not claim that the difference in *R* between the two treatments is itself statistically significant. In fact, *post hoc* application of a parametric bootstrap, estimating the distribution of differences between *R*_high_ and *R*_low_ for boldness (following Morrissey *et al.* [46]), generates approximate 95% CI of −0.26 to 0.99 that clearly span zero. By contrast to boldness, activity was significantly repeatable for both high (*R* = 0.57 ± 0.21 s.e.; LRT, $\chi_{1}{\;}^{2}=4.45$, *p* = 0.017) and low diet males (*R* = 0.52 ± 0.20 s.e.; LRT, $\chi_{1}{\;}^{2}=6.09$, *p* = 0.007). As in the case of boldness, a parametric bootstrap of differences in *R* between diets for activity generated approximate 95% CIs that overlapped zero [−0.52, 0.62].

**Table 1.** Results of Wald *χ*^2^ tests for the fixed effect of diet (compared against a *χ*^2^ distribution with 1 d.f.) on each trait in univariate linear mixed-effects models. Models for boldness and activity also included a fixed effects of assay number.

trait	$\chi_{1}{\;}^{2}$	*p*-value
boldness	0.75	0.387
activity	1.72	0.190
sperm number	0.07	0.793
sperm velocity	0.54	0.461

### 3.2. Multivariate analyses

**Figure 1.**
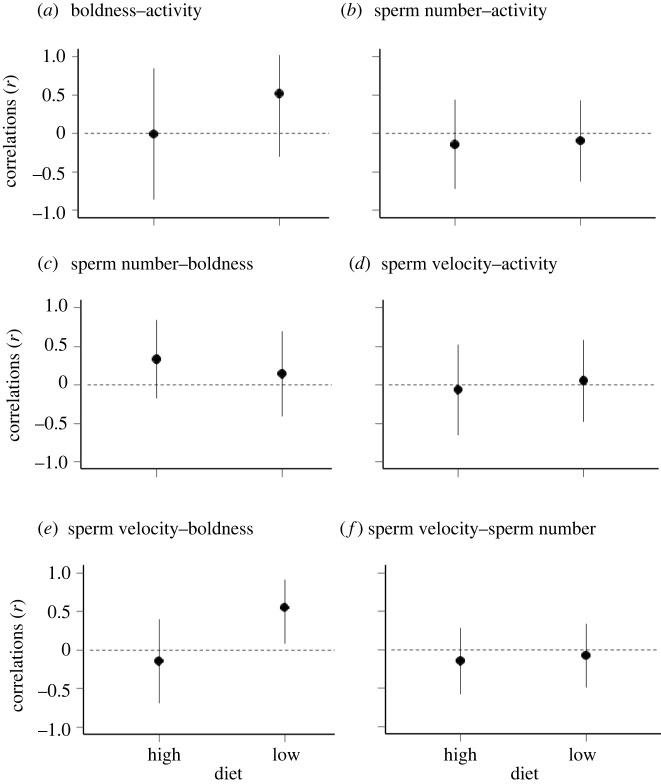
Correlations (*r*) for each pairwise combination of traits (boldness, activity, sperm number and sperm velocity) within each diet treatment, calculated using multivariate mixed-effects models in ASreml-R. Bars represent approximate 95% confidence intervals. Dotted horizontal lines are at *r* = 0. *r* and approximate 95% CIs are bounded at −1 and +1.

In agreement with the univariate analyses, there was no significant mean effect of diet on the multivariate phenotype (*F*_4_ = 0.76, *p* = 0.560), while boldness was marginally higher (*F*_1_ = 3.71, *p* = 0.061) and activity significantly higher (*F*_1_ = 13.34, *p* = 0.001) in the first assay repeat than the second. There was no evidence of any significant overall associations among traits in the full dataset (LRT comparing the full multivariate model to one with all among-individual trait correlations set to zero; *G*^2^ = 4.15, d.f. = 6, *p* = 0.657; see also electronic supplementary material, tables S2 and S3). When comparing the pairwise trait correlations from each diet treatment, the bootstrapped differences between treatments overlapped zero for all pairwise correlations (table 2 and figure 1). However, the correlation between sperm velocity and boldness was significantly higher than zero in the low diet treatment (LRT comparing a bivariate model on sperm velocity and boldness in the low diet, allowing a correlation between the two traits, to a model with the sperm velocity–boldness correlation set to zero; *G*^2^ = 4.07, d.f. = 1, *p* = 0.044), but not in in the high diet treatment (LRT; *G*^2^ = 0.28, d.f. = 1, *p* = 0.595; figure 1*e*). This suggests that bolder males had higher sperm velocity than shyer males in the low diet treatment, but not in the high diet treatment; although we note that bootstrapped differences in this correlation between the two treatments slightly overlapped zero (95% CIs = [−1.41, 0.02]). No other among-trait correlations were significantly different from zero in either treatment (figure 1; electronic supplementary material).

**Table 2.** Mean and approximate 95% confidence intervals from parametric bootstrap of the differences between pairwise trait correlation coefficients in each treatment.

correlation	mean difference	95% CIs of differences
boldness–activity	−0.52	[−1.68, 0.68]
sperm number–boldness	0.47	[−0.29, 1.22]
sperm number–activity	−0.05	[−0.83, 0.73]
sperm velocity–boldness	−0.69	[−1.41, 0.02]
sperm velocity–activity	−0.11	[−0.89, 0.67]
sperm number–sperm velocity	−0.06	[−0.65, 0.52]

## 4. Discussion

The results from this study did not support our primary hypotheses that experimental manipulation of male condition would influence the mean expression of ejaculate and behavioural traits, and the multivariate structure of covariation between personality and sperm traits. We found that mean behavioural traits (boldness and activity) were unaffected by male dietary status, although there was some evidence that variation in boldness among males (measured by repeatability, i.e. individual consistency) was reduced under low diets. Furthermore, and despite prior evidence to the contrary (see Introduction), we report that dietary restriction had no significant effect on either sperm production or sperm behaviour. Finally, our results, while not revealing the positive association between sperm production and boldness reported previously for the same population [11], did reveal an association between sperm velocity and boldness under dietary restriction. We discuss each of these findings in turn below.

Our finding that dietary restriction had no effect on mean behavioural traits did not support our hypothesis or evidence from other species that the expression of more proactive personalities, such as higher aggression, exploration, and activity, will be costly in terms of their energetic demands [47–49] and thus dependent on individual condition (see also [50]). Interestingly, a previous study on guppies conducted by Herdegen-Radwan [51] also found no effect of condition, as manipulated through a single generation of inbreeding, on either activity or boldness. A possible explanation for the lack of association between condition and personality in guppies is that displays of boldness and higher activity do not incur significantly high metabolic costs. This would be surprising, however, as proactive personality scores such as these have been linked with higher energy demands across a range of species, including Arctic char (*Salvelinus alpinus*) [47], domestic dogs (*Canis familiaris*) [52] and a marine gastropod (*Littoraria irrorata*) [53]. Further, our analyses revealed significant repeatability (i.e. individual consistency) of boldness in high diet males, but not low diet males. While the bootstrapped differences in repeatability between treatments slightly overlapped zero, the large difference in effect sizes between high and low diets provides at least qualitative support for higher consistency in boldness among high diet males. This may suggest that the maintenance of behavioural repeatability, rather than bold phenotypes *per se*, incurs metabolic costs in this species. A more focused investigation of potential physiological markers of metabolic rates in guppies should be a focus for future research, as this may provide greater clarity and precision into the energetic costs of proactive personalities in this species.

Our finding that dietary restriction did not influence any of the ejaculate traits measured in this study was unexpected and contrary to prior evidence reported for guppies. One potential factor explaining these discrepancies with prior findings is that earlier studies tended to manipulate male condition (i.e. impose dietary restriction) over a longer period (three to four months) [25,26,54] compared to the present study (one month). However, we suspect this explanation is unlikely for two reasons. First, guppies exhibit a spermatogenic cycle of approximately 36 days [29], and therefore our dietary treatment should have impacted almost all critical phases of developing sperm. Second, at least one previous study on the same population revealed a significant decline in the number, velocity and viability (% live) of sperm in food-restricted male guppies when they were treated for just one month [30]. We therefore suspect that the duration of feeding trials *per se* was not an important factor in this regard. Instead, we propose that the ontogenetic developmental stage at which dietary manipulations commence may influence the efficacy of diet restriction trials. Most prior studies investigating this question have commenced dietary trials when males first reach sexual maturity at approximately three months of age [25,26,54]. By contrast, our trials commenced when males were approximately six months of age, which matches more closely the protocol used by Devigili *et al.* [24], who also reported no significant effect of dietary restriction on either sperm number or velocity (although they did report an effect for sperm viability). One other study reporting significant reductions in sperm quality following just six weeks of dietary restriction in six-month-old males imposed a far harsher diet restriction regimen than the one used in the present study [55]. We therefore strongly suspect that the ontogenetic stage of development at which dietary manipulations commence, as well as the severity of dietary restriction, are critical factors determining the efficacy of such treatments. We are currently testing these predictions with ongoing experimental work.

Our study did not reveal the expected significant positive association between sperm production and boldness, which was reported previously in the same population used here [11]. However, it is worth noting that the pattern of covariation between sperm number and boldness reported by Gasparini *et al.* [11] was weak and only marginally significant, and thus not substantially different from the non-significant trend for such an association revealed by our study in the high-diet treatment. However, our multivariate analyses did reveal a significant positive association between sperm velocity and boldness in the low- but not high-diet treatment. Although we interpret this result cautiously—especially in the light of the low repeatability for boldness under dietary restriction—this finding has implications when we interpret *average* relationships between behavioural traits (such as boldness) and reproductive fitness that have been reported more broadly (see [1]). Our present findings suggest that such patterns may depend on the ecological context in which behaviour is observed (for a broader review on context-specific patterns of trait covariance, see [56]). Moreover, this finding of context-dependent pairwise association between boldness and sperm behaviour may have implications for patterns of selection and the speed of evolutionary processes, to the extent that both traits exhibit sufficient additive genetic (co)variance in the direction of selection (for sperm traits, see [57]). Thus, selection may favour socially dominant, aggressive or generally bold individuals when competition for resources is intense (e.g. when food is limited), and this may have implications for the reproductive success of such individuals due to their advantage during postcopulatory sexual selection. More broadly, temporal (e.g. seasonal) and/or spatial (population-level) fluctuations in environmental conditions may have implications for evolutionary processes, such as population divergence in fitness traits and/or maintenance of variation in fitness-related traits (e.g. [58]). However, an important caveat here is our finding of repeatability in boldness under high diets but not low diets. Given repeatability (individual variance) sets the upper limit for heritability of traits [59], there may be reduced potential for boldness to respond to selection under dietary stress. We acknowledge that these ideas are speculative, but our findings do open up the possibility of further investigating the potential reproductive costs and benefits exhibited by individuals that vary in boldness under different ecological contexts.

We found low repeatability (boldness) and high repeatability (activity) in the two behavioural traits considered in this study. While our estimate for boldness is generally low compared to previous personality research across a diverse range of species (see [60]), it is nevertheless within the range for similar emergence time assays reported for domestic strains of guppies [33]. Similarly, our repeatability estimate for activity is largely consistent with previous work of both feral and domestic guppies [33]. In the case of activity, we acknowledge that the relatively short interval between successive behavioural assays (1–2 days) may have inflated the value due to similar repeatable environmental conditions experienced by males across both trials, possibly generating ‘pseudo-repeatability’ for this trait (e.g. [60,61]). While our assays broadly followed the protocols adopted by other personality research on guppies, future studies might usefully explore how the duration of the interval (e.g. ranging from days to months) influences behavioural repeatability.

In conclusion, our study revealed no effect of male condition on mean behavioural traits (boldness and activity levels) or ejaculate traits, although the individual consistency of boldness was reduced under dietary stress. These unexpected findings lead us to suspect that there may be a critical developmental ‘window’ during which dietary stress most impacts fitness traits in maturing males. Furthermore, we report a context-dependent positive association between sperm velocity and boldness, which was apparent in males experiencing dietary restriction but not in those fed ad libitum*.* This latter finding suggests that environmental factors may influence the covariances between personality and sexual traits, thus potentially impacting evolutionary processes in heterogeneous environments. We advocate for further studies that explore the fine-scale physiological costs of behavioural plasticity in guppies, and those that investigate the possible evolutionary implications of changes in behavioural–sperm trait covariances across environmental gradients.

## Data Availability

The datasets supporting this article have been uploaded to the Dryad Digital Repository (https://doi.org/10.5061/dryad.00000005n) [62]. Associated R code is available on Zenodo (https://doi.org/10.5281/zenodo.6491274) and has also been uploaded as part of the electronic supplementary material [63].
